# VarEPS-Influ:an risk evaluation system of occurred and virtual variations of influenza virus genomes

**DOI:** 10.1093/nar/gkad912

**Published:** 2023-10-27

**Authors:** Chang Shu, Qinglan Sun, Guomei Fan, Kesheng Peng, Zhengfei Yu, Yingfeng Luo, Shenghan Gao, Juncai Ma, Tao Deng, Songnian Hu, Linhuan Wu

**Affiliations:** Microbial Resource and Big Data Center, Institute of Microbiology, Chinese Academy of Sciences, Beijing 100101, China; State Key Laboratory of Microbial Resources, Institute of Microbiology, Chinese Academy of Sciences, Beijing 100101, China; Microbial Resource and Big Data Center, Institute of Microbiology, Chinese Academy of Sciences, Beijing 100101, China; Chinese National Microbiology Data Center (NMDC), Beijing 100101, China; Microbial Resource and Big Data Center, Institute of Microbiology, Chinese Academy of Sciences, Beijing 100101, China; Chinese National Microbiology Data Center (NMDC), Beijing 100101, China; Microbial Resource and Big Data Center, Institute of Microbiology, Chinese Academy of Sciences, Beijing 100101, China; Chinese National Microbiology Data Center (NMDC), Beijing 100101, China; Microbial Resource and Big Data Center, Institute of Microbiology, Chinese Academy of Sciences, Beijing 100101, China; Chinese National Microbiology Data Center (NMDC), Beijing 100101, China; Microbial Resource and Big Data Center, Institute of Microbiology, Chinese Academy of Sciences, Beijing 100101, China; State Key Laboratory of Microbial Resources, Institute of Microbiology, Chinese Academy of Sciences, Beijing 100101, China; University of Chinese Academy of Sciences, Beijing 100049, China; Microbial Resource and Big Data Center, Institute of Microbiology, Chinese Academy of Sciences, Beijing 100101, China; State Key Laboratory of Microbial Resources, Institute of Microbiology, Chinese Academy of Sciences, Beijing 100101, China; Microbial Resource and Big Data Center, Institute of Microbiology, Chinese Academy of Sciences, Beijing 100101, China; State Key Laboratory of Microbial Resources, Institute of Microbiology, Chinese Academy of Sciences, Beijing 100101, China; Chinese National Microbiology Data Center (NMDC), Beijing 100101, China; Microbial Resource and Big Data Center, Institute of Microbiology, Chinese Academy of Sciences, Beijing 100101, China; CAS Key Laboratory of Pathogenic Microbiology & Immunology, Institute of Microbiology, Chinese Academy of Sciences, Beijing 100101, China; Microbial Resource and Big Data Center, Institute of Microbiology, Chinese Academy of Sciences, Beijing 100101, China; State Key Laboratory of Microbial Resources, Institute of Microbiology, Chinese Academy of Sciences, Beijing 100101, China; University of Chinese Academy of Sciences, Beijing 100049, China; Microbial Resource and Big Data Center, Institute of Microbiology, Chinese Academy of Sciences, Beijing 100101, China; State Key Laboratory of Microbial Resources, Institute of Microbiology, Chinese Academy of Sciences, Beijing 100101, China; Chinese National Microbiology Data Center (NMDC), Beijing 100101, China

## Abstract

Influenza viruses undergo frequent genomic mutations, leading to potential cross-species transmission, phenotypic changes, and challenges in diagnostic reagents and vaccines. Accurately evaluating and predicting the risk of such variations remain significant challenges. To address this, we developed the VarEPS-Influ database, an influenza virus variations risk evaluation system (VarEPS-Influ). This database employs a ‘multi-dimensional evaluation of mutations’ strategy, utilizing various tools to assess the physical and chemical properties, primary, secondary, and tertiary structures, receptor affinity, antibody binding capacity, antigen epitopes, and other aspects of the variation's impact. Additionally, we consider space-time distribution, host species distribution, pedigree analysis, and frequency of mutations to provide a comprehensive risk evaluation of mutations and viruses. The VarEPS-Influ database evaluates both observed variations and virtual variations (variations that have not yet occurred), thereby addressing the time-lag issue in risk predictions. Our current one-stop evaluation system for influenza virus genomic variation integrates 1065290 sequences from 224 927 Influenza A, B and C isolates retrieved from public resources. Researchers can freely access the data at https://nmdc.cn/influvar/.

## Graphical Abstract

**Graphical Abstract:**
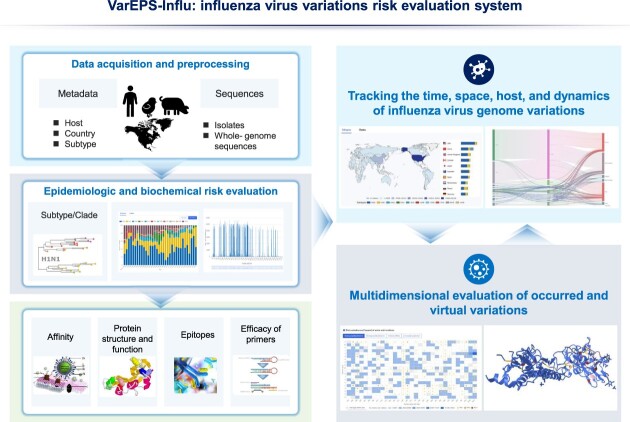

## Introduction

The influenza virus belongs to the Orthomyxoviridae family and is a negative-strand single-stranded RNA virus. It represents one of the most common respiratory pathogens affecting humans. Seasonal influenza, caused by influenza A and influenza B, results in approximately 1 billion infections, 3–5 million severe cases, and 290000 to 650000 deaths related to respiratory diseases annually, imposing a substantial public health burden [World Health Organization (WHO) statistics: https://www.who.int/news-room/fact-sheets/detail/influenza-(seasonal)].

The influenza virus genome exhibits high variability, enabling it to evade host immune responses, leading to persistent epidemics and periodic pandemics. Antigenic drift and antigenic shift (1) are the main forms of genomic variation that can impact the antigenicity (2,3), drug resistance (4), virulence (5) and host range (6–8) of the virus, significantly affecting human health. Consequently, a comprehensive understanding of influenza virus genomic variation is crucial for influenza prevention and control.

Over the past few decades, scientists have made significant progress in identify key variation sites within the influenza virus genome and their implications on the virus' biological characteristics. Studies by Hensley *et al.* demonstrated that single point mutations can increase the hemagglutinin receptor's binding affinity and drive antigenic drift in influenza A virus (2). Additionally, studies by Koel *et al.* revealed that substitutions at seven positions of the human A/H3N2 seasonal influenza virus near the HA receptor binding site lead to major antigenic changes in influenza virus evolution (3). The H275Y amino acid mutation in neuraminidase (NA) contributes to Oseltamivir resistance in the H1N1 subtype (4). Furthermore, single amino acid substitutions in PB2 and HA play vital roles in influencing influenza virulence (5). Notably, mammalian adaptative mutations in PB2, such as E627K and D701N, significantly enhance the replication, pathogenicity, and transmissibility of avian influenza viruses in mammals (6–8).

Despite significant advancements in understanding some key variation sites, these achievements are primarily based on experimental results and lineage evolution studies. However, the number of studied mutations remains limited compared to the vast influenza virus genomes, particularly concerning those mutations that have not yet occurred. Genomic variation can affect various aspects of proteins, including their physical and chemical properties, primary, secondary and tertiary structures, receptor affinity, antibody binding capacity and antigenic epitopes. Numerous tools and methods have been developed to assess these effects, such as PROVEAN (9), Saambe-3D (10), BepiPred (11) and ElliPro (12). However, these tools generally focus on individual aspects and lack integration with spatiotemporal dynamics of variations. Consequently, there is a critical need for a comprehensive, dynamic and multi-dimensional understanding of influenza virus genome variation.

To address this need, we developed VarEPS-Influ, a one-stop analysis system for influenza virus genome variation. VarEPS-Influ integrates spatiotemporal monitoring, lineage analysis, mutation identification and risk evaluation. We comprehensively assess the effects of variation on physical and chemical properties, primary, secondary, and tertiary structures, receptor affinity, antibody binding capacity and epitopes, combining these evaluations with epidemiological data to provide comprehensive evaluation results. Notably, our system evaluates both observed mutations and predicts the potential impact of ‘virtual mutations’ that have not yet occurred, thus offering crucial information and strategies for the prevention and control of influenza viruses.

## Materials and methods

### Data sources and data processing

**Figure 1.**
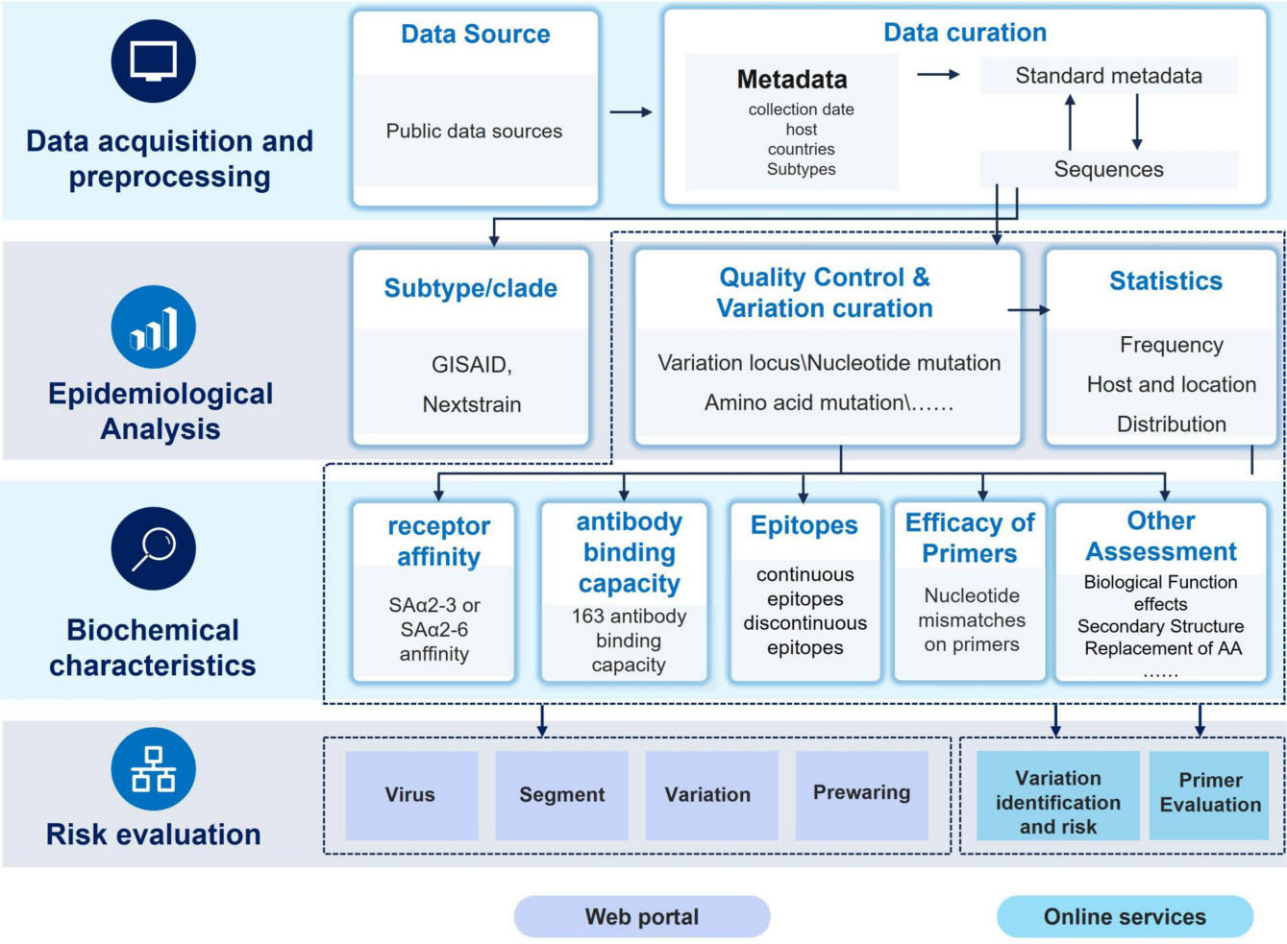
Schematic representation of data processing and online analysis services in VarEPS-Influ.

We obtained data from Genbank (13) and extracted 902 036, 161 269 and 1 985 sequences for Influenza A virus, Influenza B virus, and Influenza C virus, respectively by the end of August 2023. After removing the sequences <150 bp, we using Mafft (15) to mapped each sequence against the segment references to identify mutations and deletions. Reference sequences for A subtype were obtained from the Influenza Virus Resource at NCBI (14), while those for influenza B (Assembly ID: GCF_000820495.2) and C (Assembly ID: GCF_000856665.10) subtypes were obtained from the Genbank which were indicated as ‘ICTV (International committee on Taxonomy of Virus) species exemplar’. We employed the same site-numbering scheme as the reference genome to generate lists of nucleotide variants and amino acid variants. Subsequently, we examined each mutation according to the aspects outlined in Figure 1.

### Predict the effects of single amino acid mutation on protein–protein interaction (PPI)

Using Saambe-3D (10), we predicted the effects of single amino acid mutations on protein-protein interactions, including changes in binding free energy. Mutation types were classified as destabilizing (ΔΔ*G* > 0), stable (ΔΔ*G* < 0), highly destabilizing (ΔΔ*G* > 1.5), or highly stable (ΔΔ*G* < –1.5). Additionally, we predicted the influence of single amino acid variations on the affinity of influenza virus to neutralizing antibodies and sialic acid receptors α2,3/α2,6.

To assess the effects of mutations on binding free energy, we downloaded 163 PDB files of influenza virus binding to neutralizing antibodies, 77 PDB files of influenza virus binding to sialic acid receptor α2,3, and 68 PDB files of influenza virus binding to sialic acid receptor α2,6 from the PDB database (16). The list of PDB files can be found at https://nmdc.cn/influvar//about. Subsequently, we used Saambe-3D software to calculate the change in binding free energy for all observed and unobserved amino acid changes. A decrease in neutralizing antibody affinity greater than (ΔΔ*G* > 1.5) and an increase in sialic acid receptor binding capacity greater than (ΔΔ*G* < 0) were used as risk thresholds. We divided the overall risk level into three levels (1, 2, 3) by accumulating and averaging individual risk values.

### Assessment of the difficulty of occurrence of Nucleotide Diversity

We evaluated the ‘Nonsynonymous density’ value as a reflection of the difficulty of nucleotide diversity occurrence. This assessment involved calculating the density of synonymous mutations and nonsynonymous mutations under a sliding window. High frequency of nonsynonymous densities indicated rapidly accumulating mutations in a region, while low frequency densities suggested regions where potential selection of elimination occurred, implying long-term and stable adaptive changes in the virus (17).

### Evaluation of the risk of amino acid replacement

We utilized the PAM (18) and BLOSUM (19) matrices to assess the ‘risk of amino acid replacement’. High substitution probability of two amino acids indicates that such replacements are accepted by nature, leading to a lower risk level evaluation. For two amino acids with low substitution probability, but there is a frequent occurrence of such substitution, this may suggest that this mutation plays an important role in the survival or transmission of the virus.

### Prediction of effects on biological function of proteins

To predict the effects of amino acid variants on protein biological functions, we used the PROVEAN (9) software and set the threshold for destructiveness and neutrality at –2.5.

### Assessment of effects on secondary structure

For ‘secondary structure prediction’ of mutated proteins and comparison with published PDB files on X-ray diffraction of proteins, we employed bepipred2.0 (11) and recorded the changes.

### Prediction of effects on potential continuous and discontinuous epitopes

ElliPro (12) was used to predict ‘changes of antigen continuous epitopes’ and ‘changes of antigen discontinuous epitopes’ before and after the variation occurred.

### Evaluation of the effect on effectiveness of detection reagents

We collected 79 primers from the WHO website for PCR ‘primers efficacy evaluation’. The variant's location and amount were considered comprehensively, and risk scores were assigned if the variant occurred in the last three bases of the 3′ end of a certain primer. Additionally, the number of mutations was assessed, and a corresponding score was given based on the number of variants at the last three bases in the 3′ end. Based on these scores, the risk level of variants to PCR primers was determined.

### Analysis of epidemiological data

Epidemiological data, including sample sites, host, collection date and lineages were collected from Genbank. We generated statistical tables and graphs to analyze the frequency, location, and host distribution of the virus based on their lineage and subtypes.

## Results

### Database interface and features

The VarEPS-Influ homepage presents the global distribution of influenza A, B and C viruses, and users can directly access detailed information on isolates, genomes, nucleotide sequences, and variations through statistic numbers that are linked to corresponding lists. High-frequency variation statistics are visually summarized in graphs based on different segments, subtypes and genes. Identified high-risk variations are provided and displayed in 3D structure format.

**Figure 2.**
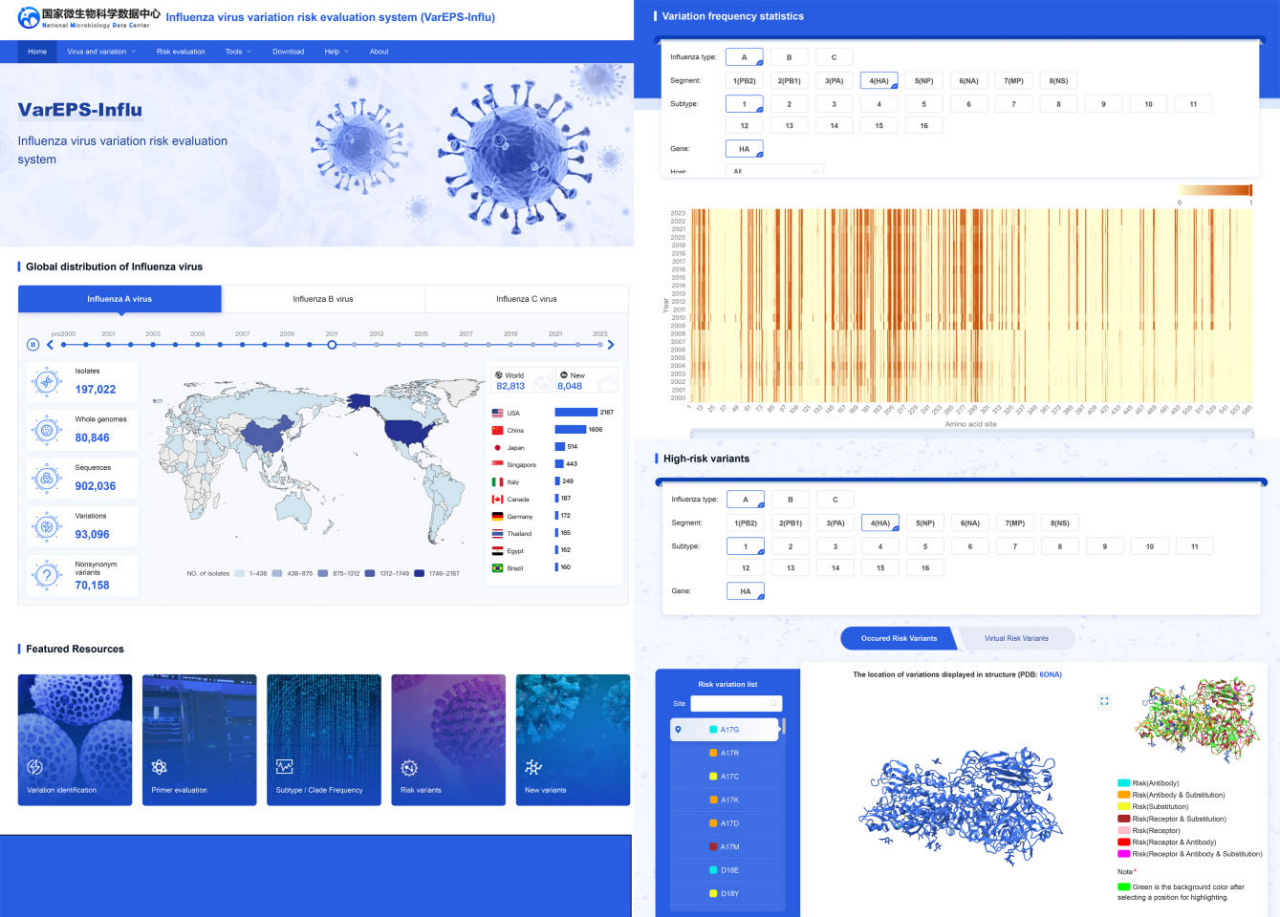
Features of the VarEPS-Influ portal.

The function pages of VarEPS-Influ consist of two main parts (Figure 2): ‘Virus and Variation’ and ‘Risk evaluation’. The ‘Virus and Variation’ section offers browsing capabilities from three perspectives: virus strains, sequences and variation sites, along with a multi-dimensional combination query function. Users can conduct searches based on various conditions, such as subtype, host, sampling area and sampling time. Query results are presented through interactive charts and tables, and a download function is available.

The ‘Risk evaluation’ section is designed to warn and display potentially dangerous mutation sites on influenza sequences. Risk sites are evaluated based on antibody affinity, receptor affinity, and the difficulty of amino acid substitution. The increased receptor affinity may lead to an increased risk of transmission of the virus. The diminished neutralizing antibody affinity could trigger immune escape. The difficulty of amino acid substitution suggests that these mutations play an important role in the survival or transmission of the virus. The assessment results together with the spatial and temporal trends of mutation sites give a comprehensive understanding on the risks and implications of viral mutations. The page showcases risk evaluation displays of dangerous sites from three perspectives: observed mutations, virtual mutations (yet to occur), and annual new mutations. A usage case is provided in supplementary 1.

### Online analysis tools

The ‘Tools’ section integrates two online tools: ‘Variation identification’ and ‘Primer Evaluation’. The ‘Variation Identification’ tool allows users to identify and assess variation sites in influenza genome sequences. Users input the sequence in FASTA format, which undergoes pre-processing and filtering before being compared with the reference sequence library to identify variation sites. The tool provides multi-dimensional evaluation results of neutralizing antibody binding ability, receptor α2,3 affinity, receptor α2,6 affinity, difficulty of amino acid substitution, and the effect of mutations on proteins.

The ‘Primer Evaluation’ tool enables users to assess the impact of genomic variation on the detection of primers. By inputting upstream and downstream primer sequences and probe sequences, the tool generates information on subtypes, clades, and viruses that affect detection effectiveness in the database, along with the location of variant sites on primers and probes.

## Data content and analysis

**Figure 3.**
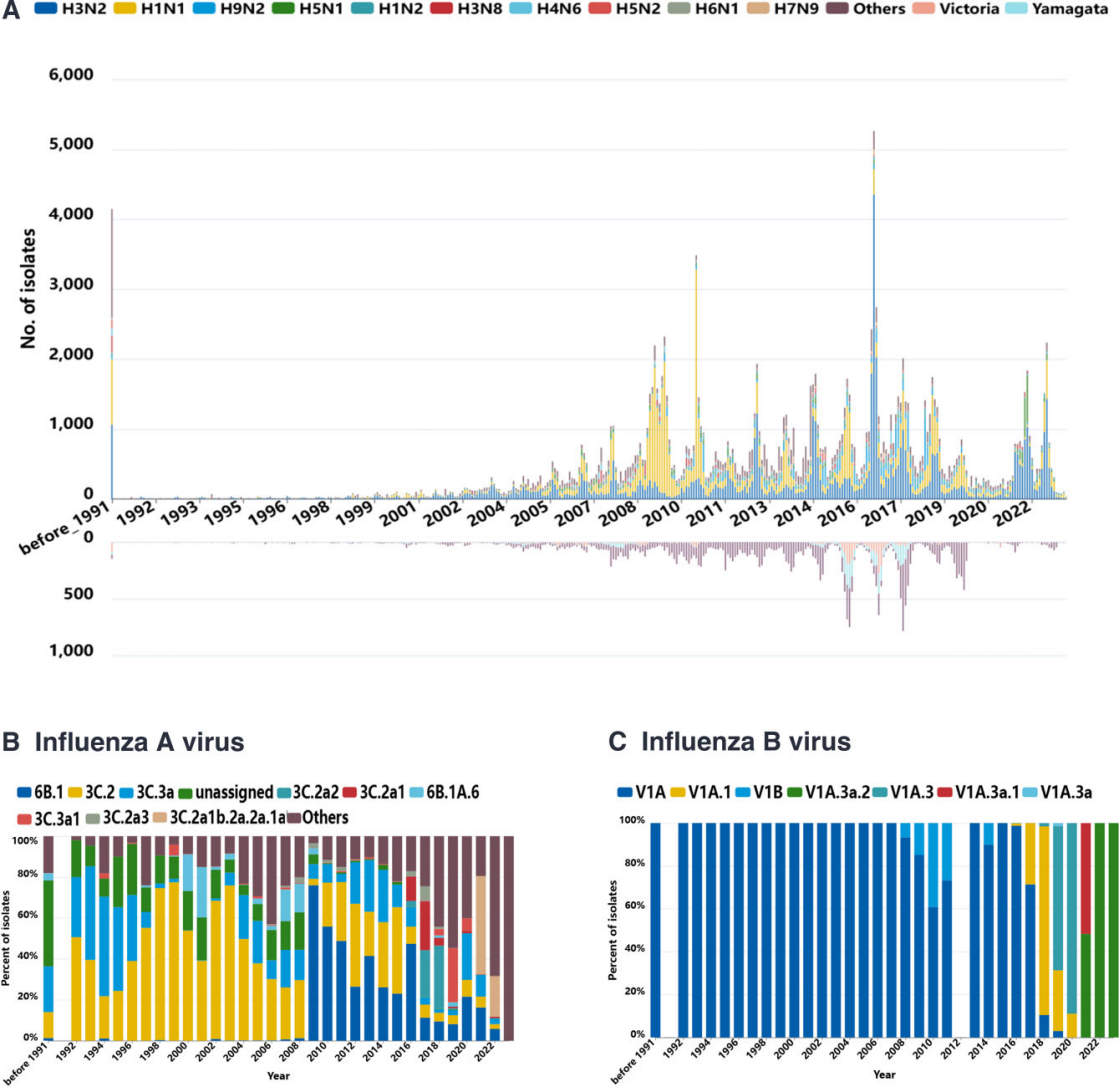
The distribution of virus subtypes of influenza A and B from 1990 to 2023. (**A**) Distribution of influenza isolates by subtypes. The upper axis indicates the distribution of subtypes of influenza A, while the lower axis indicates the distribution of subtypes of influenza B. (**B**) Distribution of influenza A isolates by clades. (**C**) Distribution of influenza B isolates by clades.

We conducted an analysis of influenza virus subtype and lineage variations over a 34-year period, from 1990 to the present (Figure 3). Overall, the number of influenza virus sequences has steadily increased over the years, with a sharp surge during the 2009 pandemic and a subsequent decline during the COVID-19 epidemic in 2020 and 2021. Influenza A virus sequences showed dominance of the H3N2 subtype in 27 out of 34 years, while the H1N1 subtype led in the remaining 7 years (Figure 3A). The 2009 H1N1 pandemic, initiated by lineage 6 B.1 (Figure 3B), resulted in an increased proportion of H1N1 subtypes from 2009 to 2013 (20). Influenza B virus data volume remained small over the past 34 years and was mainly dominated by the V1A lineage. The proportion of influenza B virus sequences relative to influenza A virus sequences fluctuated between 4% and 20% before 2015. However, from 2016 to 2020, the proportion of influenza B virus sequences continued to rise, reaching its peak at 34.32% in 2020, driven by the V1A.3 lineage of influenza B virus (Figure 3C). It should be noted that in time series data, sample sampling bias may lead to inaccurate description of time trend. For example, the decline in the number of influenza virus sequences during the COVID-19 pandemic may be due to the impact of restrictions on sample collection. Close attention should be paid to the time distribution of the data, the number of samples and other factors that may be caused by sampling bias’.

The regional distribution analysis revealed that H3N2 and H1N1 are the predominant subtypes of influenza A virus worldwide, with different countries exhibiting distinct influenza virus subtype distributions. For instance, H9N2, which is prominent in China, is primarily distributed in Asia (90.29%) and to a lesser extent in Africa, North America and Europe. Furthermore, there are regional variations in the host origins of different subtypes. For example, the H9N2 virus has only been found in human host samples in Asia and Africa.

**Figure 4.**
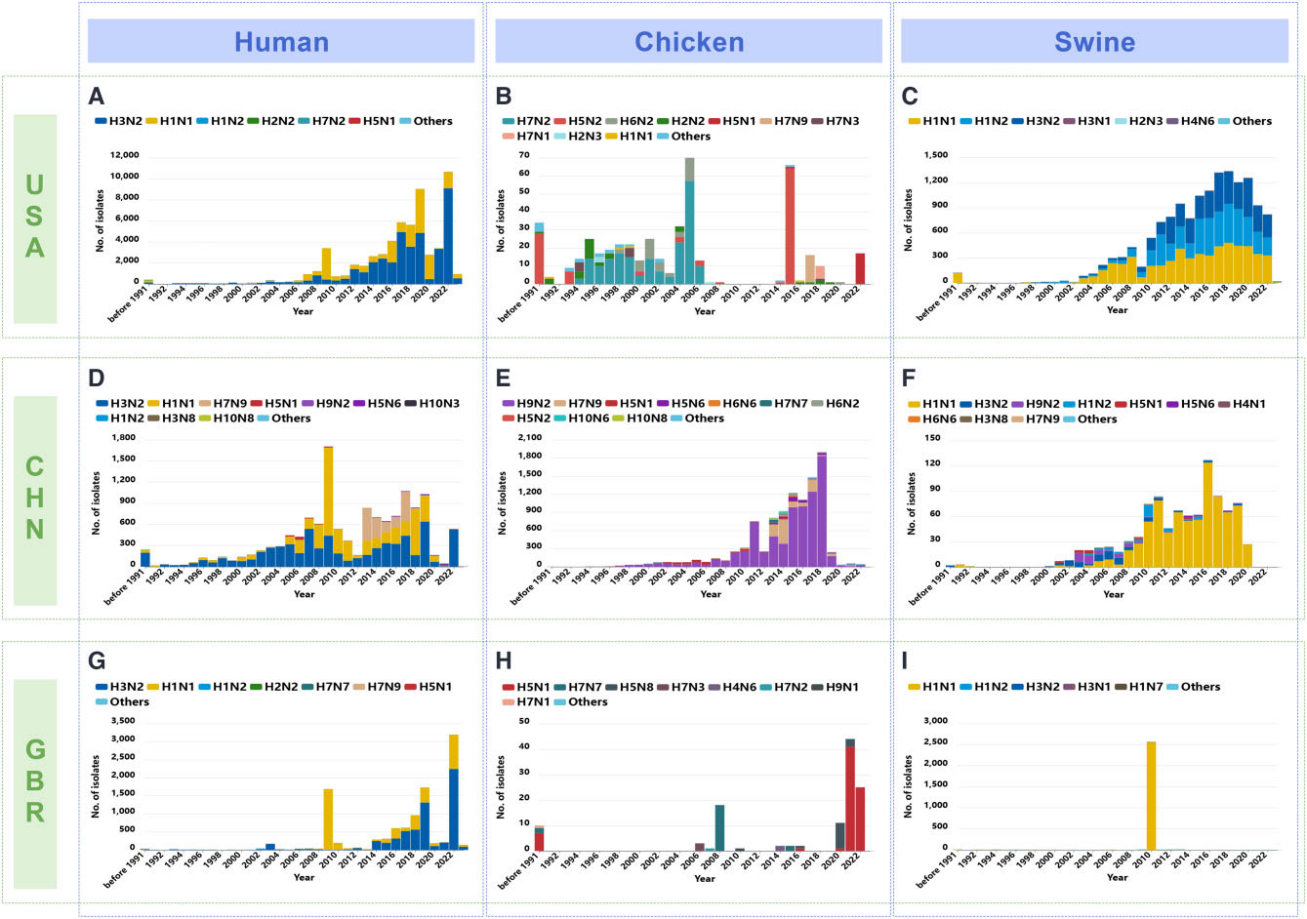
Annual trends of flu subtypes in various hosts and countries. This chart presents a comparison of annual trends of flu subtypes across various hosts and countries. Each horizontal grouping represents a different country, and each vertical grouping indicates a different host. (A–C) Influenza subtype prevalence in the United States for (**A**) human hosts, (**B**) chicken hosts and (**C**) swine populations. (D–F) Influenza subtype prevalence in the China for (**D**) human hosts, (**E**) chicken hosts and (**F**) swine populations. (G–I) Influenza subtype prevalence in the United Kingdom for (**G**) human hosts, (**H**) chicken hosts and (**I**) swine populations.

There are also notable variations in the annual epidemic trends of influenza viruses among different hosts in different countries (Figure 4). For instance, considering the top three countries with the largest number of sequences, namely the United States, China and the United Kingdom, the H1N1 and H3N2 subtypes exhibit wide distribution and robust persistence, while other influenza subtypes are primarily confined to specific regions and display weaker epidemic persistence. The proportion of influenza subtypes also differs significantly between different countries, likely influenced by factors such as geography, climate and population mobility (21). Moreover, substantial variations in the proportion of influenza lineages can be observed among different hosts within the same country, possibly linked to host species, ecological environment and lifestyle (22). As an example, the H7N9 influenza subtype appears to be region-specific, being concentrated solely in China and not causing a global epidemic (Figure 4D–F) (23).

**Figure 5.**
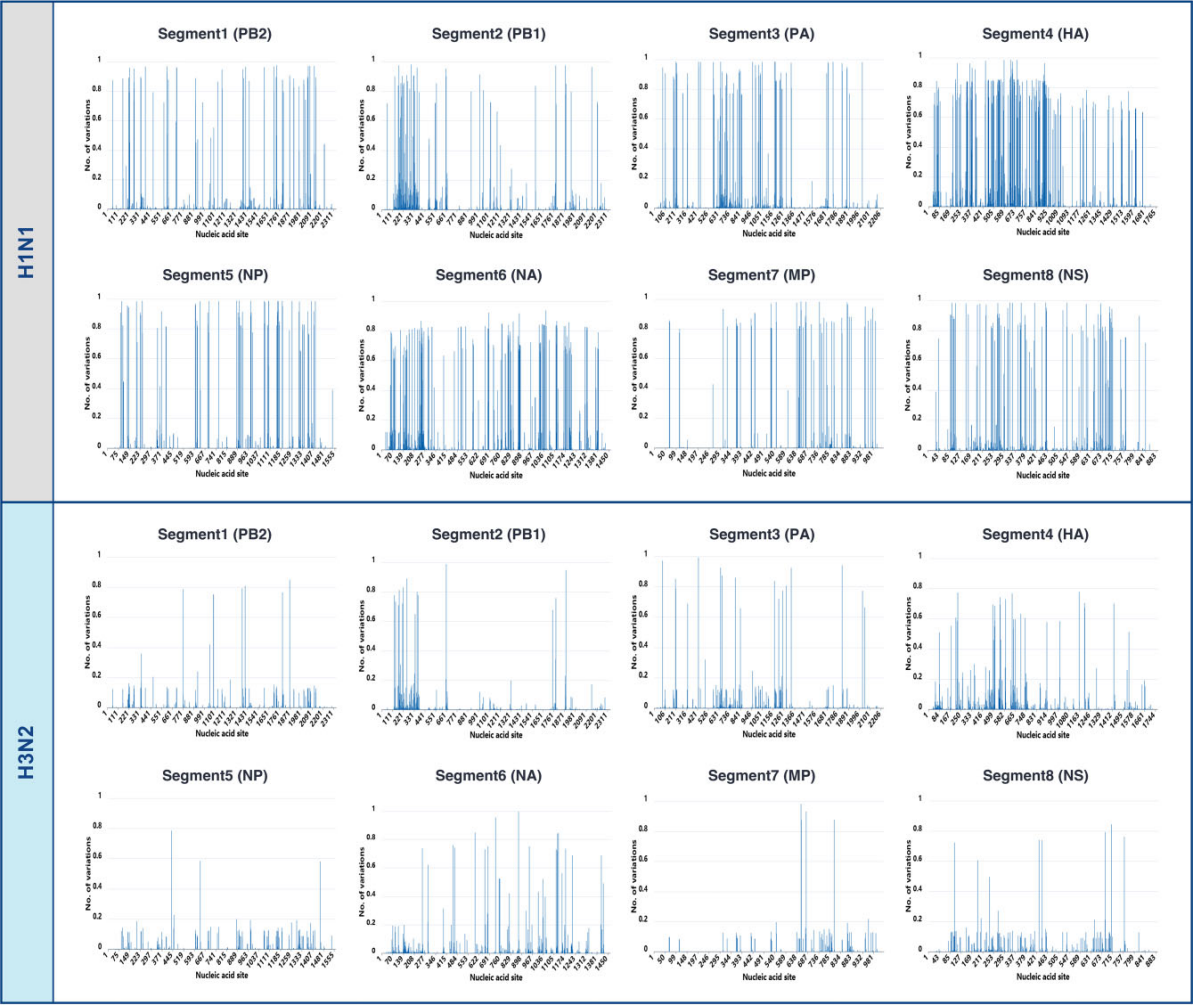
Nonsynonymous mutations frequencies in H1N1 and H3N2 influenza's eight RNA segments.

**Figure 6.**
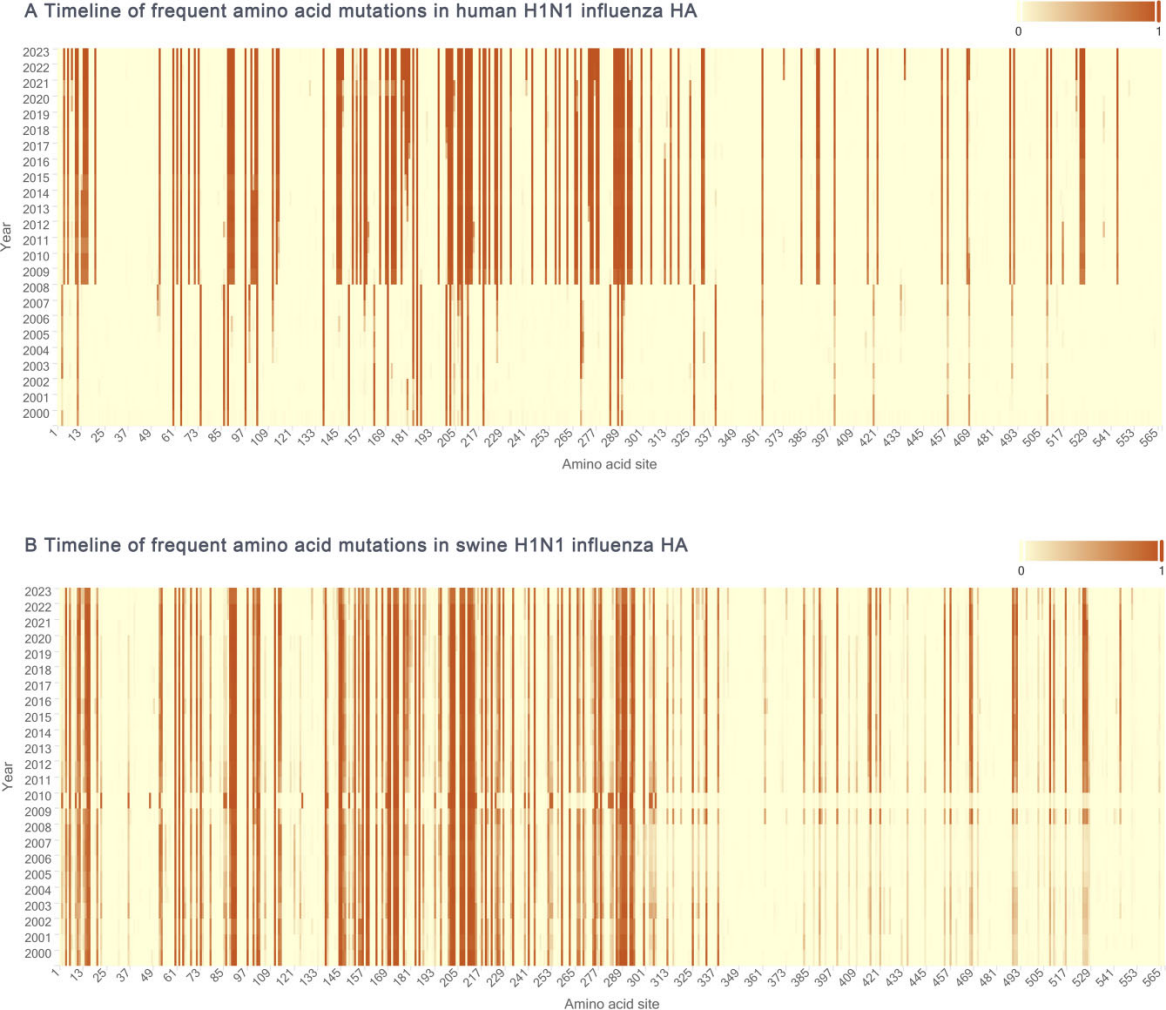
Timeline of frequent amino acid variations in human and swine H1N1 influenza HA. (A, B) Timeline of frequent amino acid mutations in (**A**) human H1N1 influenza HA and (**B**) swine H1N1 influenza HA.

**Figure 7.**
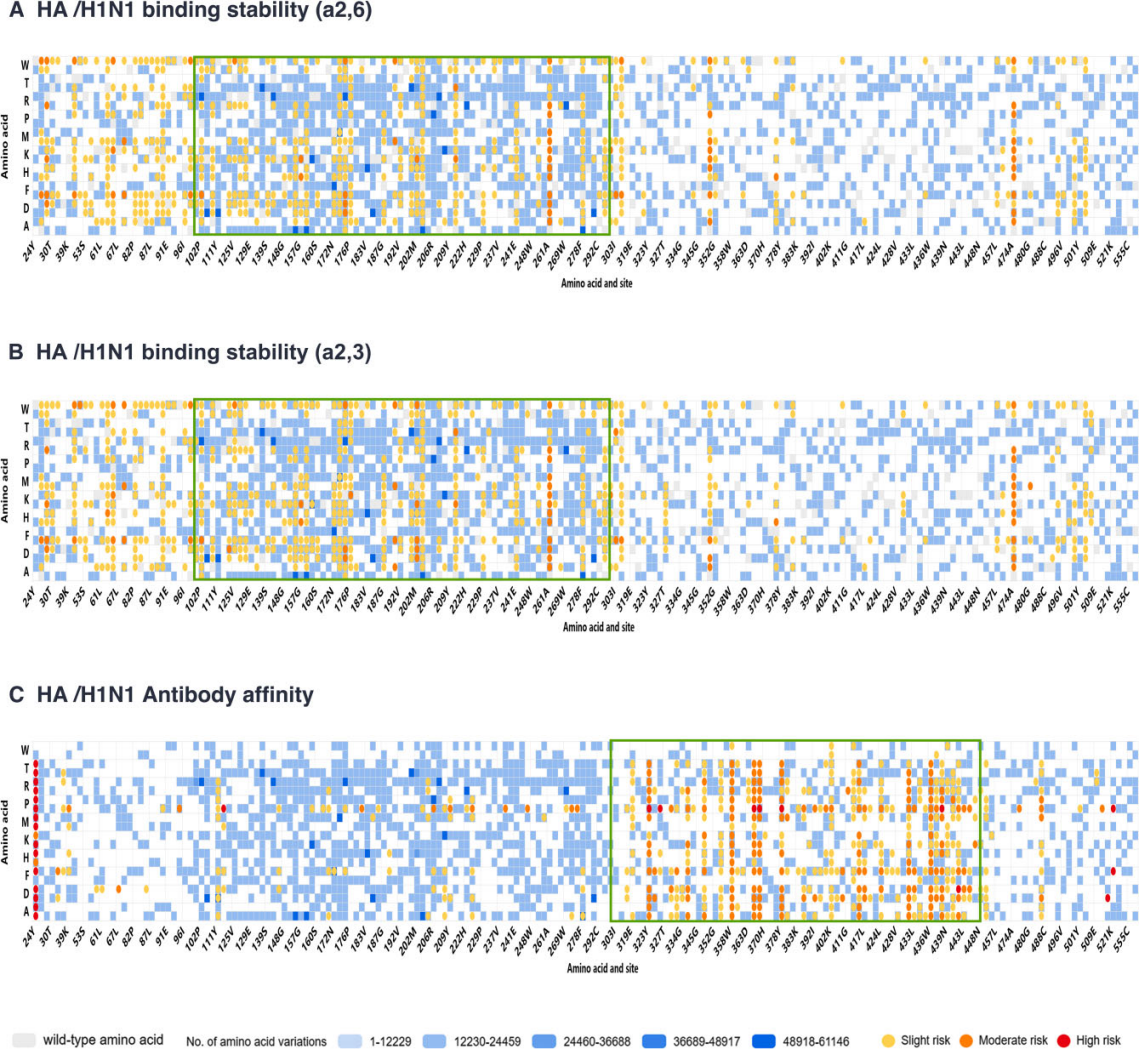
Multidimensional risk assessment of single amino acid mutations in the H1N1 influenza virus HA protein. (A–C) Evaluation of the risk of H1N1 HA variations on (**A**) SA26 receptor binding stability, (**B**) SA23 receptor binding stability and (**C**) antibody affinity. The green box is the area with high risk sites.

From 1 065 290 influenza virus sequences, encompassing A, B and C types, we identified a total of 119 245 genomic variation sites, of which 83 725 were nonsynonymous variation sites. The majority of nonsynonymous mutations were distributed in HA (40.37%), NA (24.94%). Nonsynonymous mutations distribution patterns are similar across different influenza subtypes. In the H1N1 lineage, 21.32% nonsynonymous mutations are concentrated in the HA protein, 15.95% in the NA protein. Similarly, in the H3N2 lineage, 21.42% are concentrated in the HA protein, 14.93% in the NA protein (Figure 5). We further conducted a statistical analysis of the dynamics of high-frequency mutation sites for each influenza virus subtype. Taking the HA protein of the H1N1 influenza subtype as an example, we explored the temporal variation of high-frequency mutation sites in human and pig hosts since 2000 (Figure 6). The receptor-binding domain of the HA protein of the H1N1 subtype is located in the amino acid sequence 125–251 (24), and the high-frequency variations we detected were primarily concentrated in the amino acid positions 100–300, roughly consistent with the receptor-binding domain's location. Our findings indicate that 2009 marks a crucial watershed, as numerous unique mutation sites previously limited to porcine strains began to be detected in human strains and became fixed. Concurrently, some unique mutation sites that were frequently found in human strains significantly decreased after 2009. Since 2021, the frequency of three variable sites in the HA protein of the human H1N1 subtype has shown a rapid upward trend at amino acid positions 241, 276 and 435, respectively. Notably, VarEPS-Influ identifies 12 amino acid variants at position 241 as having the potential risk of enhancing receptor affinity, and four of these variants have not yet occurred, denoted as virtual variants, warranting close attention (Figure 7A).

We performed a multidimensional evaluation of all 19 possible amino acid variants at all 26026 amino acid positions in the reference sequences within this database, comprising 92676 real variants and 401818 virtual variants. Taking the HA protein of the H1N1 subtype as an example, we assessed the effect of single amino acid variations on the affinity of influenza viruses to the sialic acid receptors α2,3 and α2,6 (Figure 7A, B). Additionally, we evaluated the effect of individual amino acid variations on the binding of influenza to 163 neutralizing antibodies (Figure 7C). As illustrated, the risk sites for receptor affinity are predominantly concentrated in amino acids 100–300, close to the receptor binding region, which is consistent with previous studies (2,3).

In addition, the sites predicted by the system with significant effects on the binding free energy of the neutralizing antibody are not only concentrated in the receptor-binding region of the HA1 polypeptides, but also in a specific region (between amino acid positions 300 to 450 of the HA fragment) of the HA2 polypeptides. For the presence of nonsynonymous mutations, HA2 polypeptides is relatively conserved compared with HA1 polypeptides (Figure 5). Previous studies suggested that subtype-specific neutralizing epitopes on the HA1 polypeptides and neutralizing epitopes on the HA2 polypeptides (25,26) could be potential candidates for the development of subtype-specific and universal vaccines against influenza viruses, respectively. As a result, high risk sites for neutralizing antibodies provided by the system provide potential vaccine design directions, although the biological mechanisms of these findings need further experimental confirmation.

Notably, previous research by Hensley *et al.* demonstrated that a single point mutation in the HA protein can lead to an increase in the hemagglutinin receptor's affinity, driving influenza A virus antigenic drift (2). Moreover, Koel *et al.* reported that major antigenic changes during influenza virus evolution result from the replacement of seven specific positions of the HA receptor binding site (3). In our multidimensional evaluation, we analyzed these seven key variable sites (i.e. sites 145, 155, 156, 158, 159, 189 and 193 of the HA protein). The results revealed that five of the seven sites (HA proteins 145, 155, 158, 159 and 193) had an affinity risk for receptor α2,6, whereas the variant at position 189 showed a risk for neutralizing antibody binding. The specific mechanisms and biological basis of these findings warrant further verification through additional experiments.

## Discussion

The genomic variations observed in influenza viruses present a significant challenge to global public health, impacting virus transmission, virulence, drug resistance, and host range, thereby influencing the overall epidemic pattern and severity of influenza (2–8). Consequently, a comprehensive understanding and effective prediction of influenza virus genomic variation are imperative for successful prevention and control measures. At present, there are several important influenza databases in the world, including NCBI’s Influenza Virus Database (14), GISAID database (27) and BV-BRC’s Influenza Database (28). The Influenza Virus Database of NCBI and GISAID database don’t focus on the risk evaluation of variation sites. The Influenza Research Database (IRD) (29), which has now been merged into BV-BRC, had functions related to mutations. However, these functions are no longer available in the new BV-BRC influenza database.

In this study, we have successfully developed VarEPS-Influ, a comprehensive one-stop analysis system for influenza virus genome variation. The system integrates lineage analysis, mutation identification, and risk assessment, allowing for a multi-dimensional evaluation of real and virtual variations. This approach, combined with spatiotemporal dynamics, enables the prediction of phenotypic changes resulting from mutations, providing essential data for the formulation of effective prevention and control strategies. By providing valuable information to experimental teams studying the underlying mechanisms, our system aids in narrowing down the scope of experiments. Continuous feedback from experimental results allows us to refine and improve the multi-dimensional evaluation system, enhancing its accuracy and practicality.

However, current understanding and predictive capabilities remain limited due to the complex and dynamic nature of influenza virus genome variation. It's difficult to provide a single risk assessment score for entire influenza virus sequence or a lineage. Although the digital evaluation results obtained through our multi-dimensional approach create a comprehensive characterization of each variation site, enabling to construct vector matrices and artificial intelligence models, more experiment data serving as training sets are necessary. We have previously applied a similar approach successfully in the construction of the SARS-CoV-2 Variations Evaluation and Pre-warning System (VarEPS) (30). Moving forward, our next step will involve further development and optimization of artificial intelligence models, leveraging the multi-dimensional parameters evaluated by VarEPS-Influ to enhance our predictive and control capabilities for influenza viruses. In summary, our study provides a novel perspective and valuable tool for investigating influenza virus genome variation. We anticipate that VarEPS-Influ will play a vital role in advancing influenza virus research and enhancing public health practices.

## Supplementary Material

gkad912_Supplemental_File

## Data Availability

There are no access restrictions for academic use of the platform. Access to VarEPS-Influ is freely available at https://nmdc.cn/influvar/.
