# Supplementary material for: VarEPS-Influ:an risk evaluation system of occurred and virtual variations of influenza virus genomes
Source: Nucleic Acids Res. 2023 Oct 27;52(D1):D798–807. doi: 10.1093/nar/gkad912 (PMC10767863; doi:10.1093/nar/gkad912)
Supplement: gkad912_Supplemental_File [file gkad912_supplemental_file.docx]

**Supplementary1. Usage Case**

This database combines epidemiological analysis with multidimensional risk assessment of mutation sites, and can be used in multiples ways by users with different research purposes. The use of the database is demonstrated through a case study exploring the effects of host specific sites on receptor affinity and antigenicity.

**Steps and results**

**Step1: Identify rapidly growing sites of variation and compare different hosts:**

First of all, we compared the time trends of human H1N1 and swine H1N1 mutation sites through the frequency heat map (https://nmdc.cn/influvar/) in the middle of the home page. It is noteworthy that three sites 241, 276 and 435 of HA protein have been persistent in swine H1N1, of which 241 loci account for a relatively high proportion of swine data, ranging from 50% to 90%. It is worth noting that these three loci have seldom been detected in human data before 2021. However, in the past two years, their proportion has risen rapidly, and in 2023, the proportion has exceeded 95%.


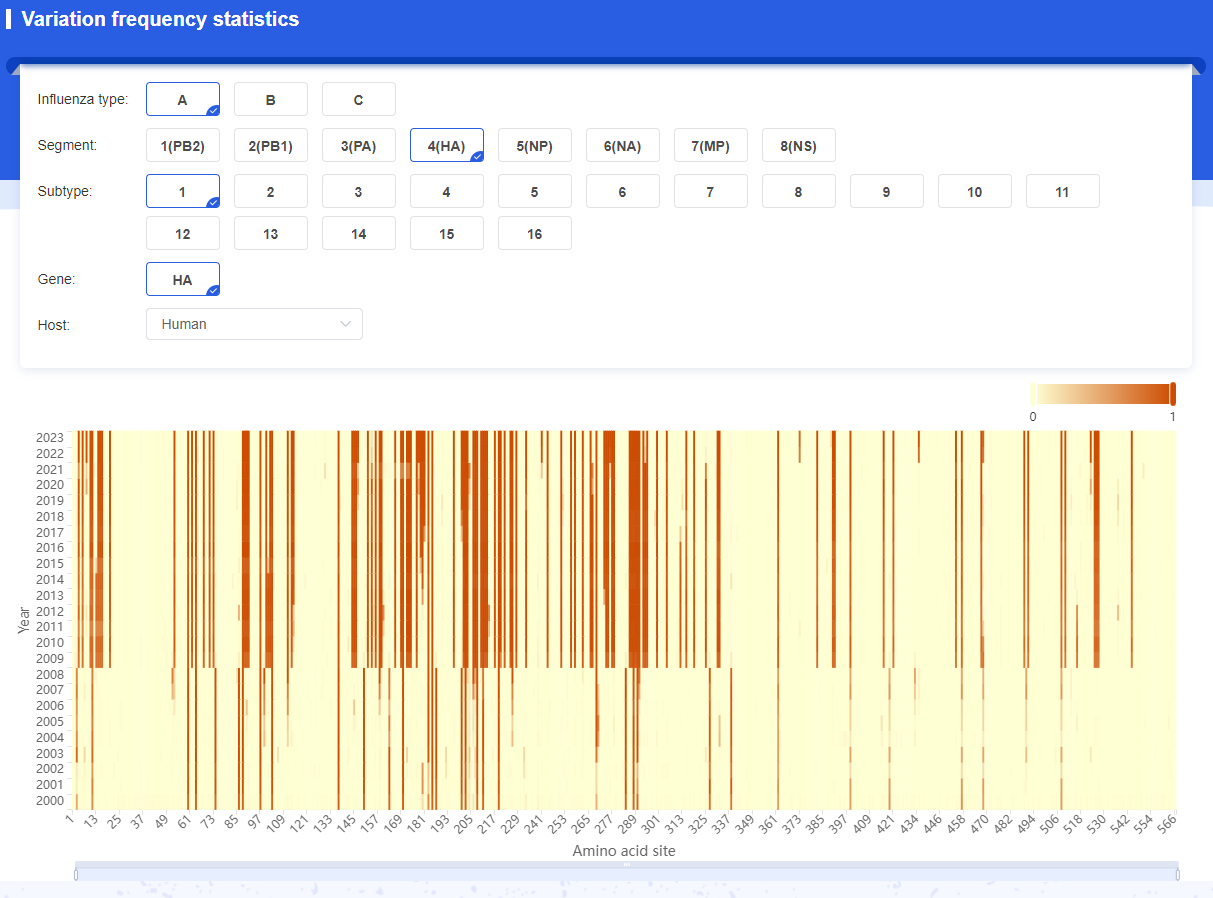


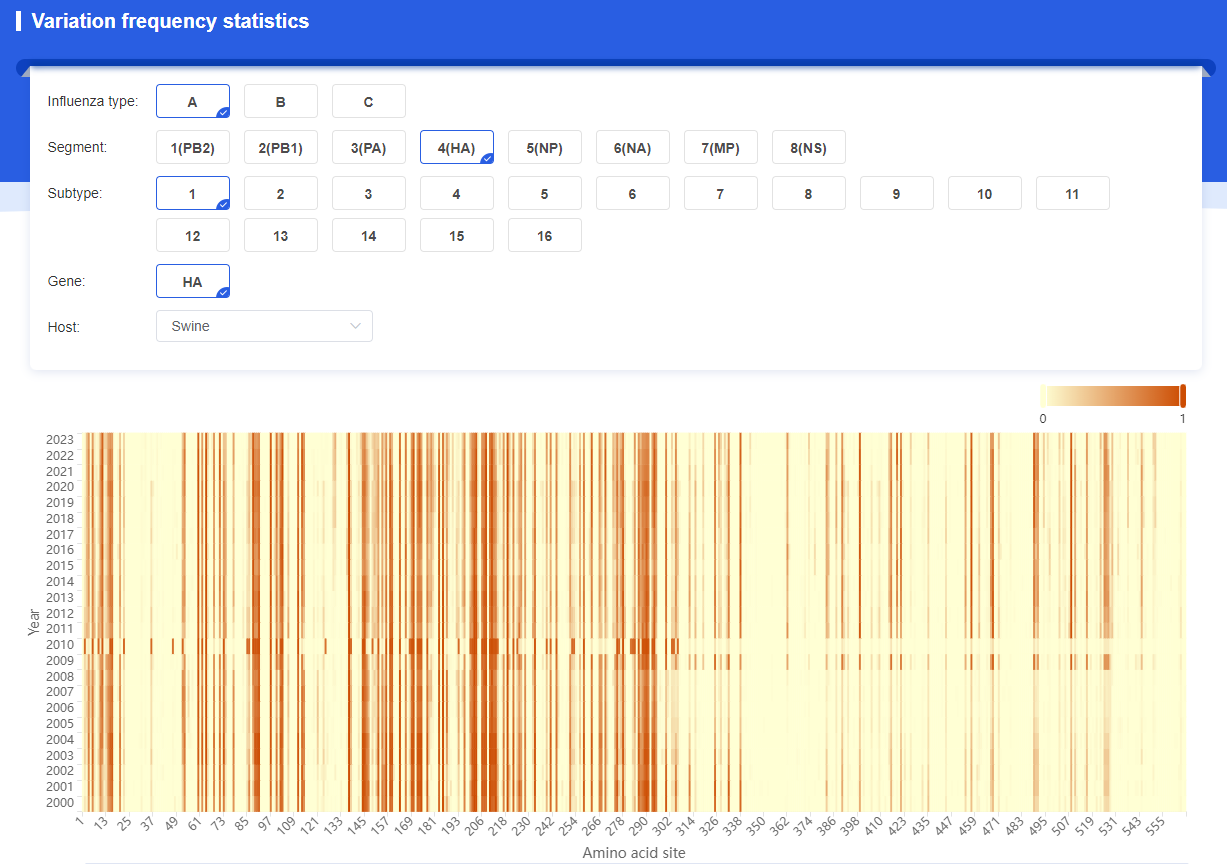


**Step2. Risk evaluation results on 3D structure Display:**

We went to the 3D structure display area of risk loci in the middle of the home page, retrieve the above three loci, and check the structural characteristics of the loci and the results of multi-dimensional risk assessment. Of these, locus 241 is predicted to have multiple dimensions of risk.


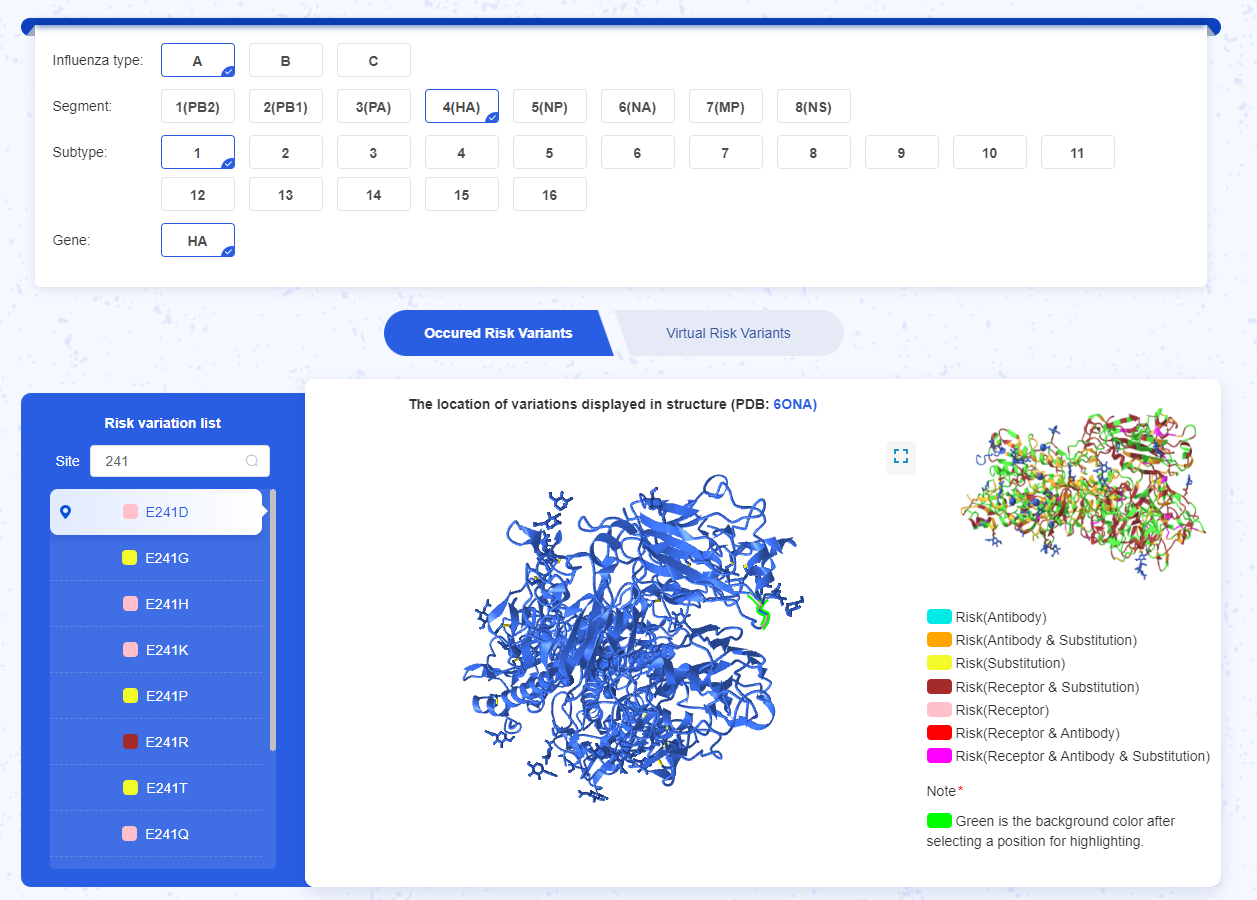


**Step3. Multi-dimensional risk assessment of sites:**

Next, we could have a comprehensive understanding of the multi-dimensional evaluation of all amino acid variations at the 241 site. On the “Risk evaluation” page, users can directly input amino acid sites, and obtain the multi-dimensional evaluation results of 241 sites, including the mutations that have occurred and the virtual mutations that have not occurred. The assessment results showed that some amino acid variations had high risks. In addition, the “Risk evaluation” page also displays the lineage distribution, host distribution, temporal distribution and other information of the sequence carrying the variation.


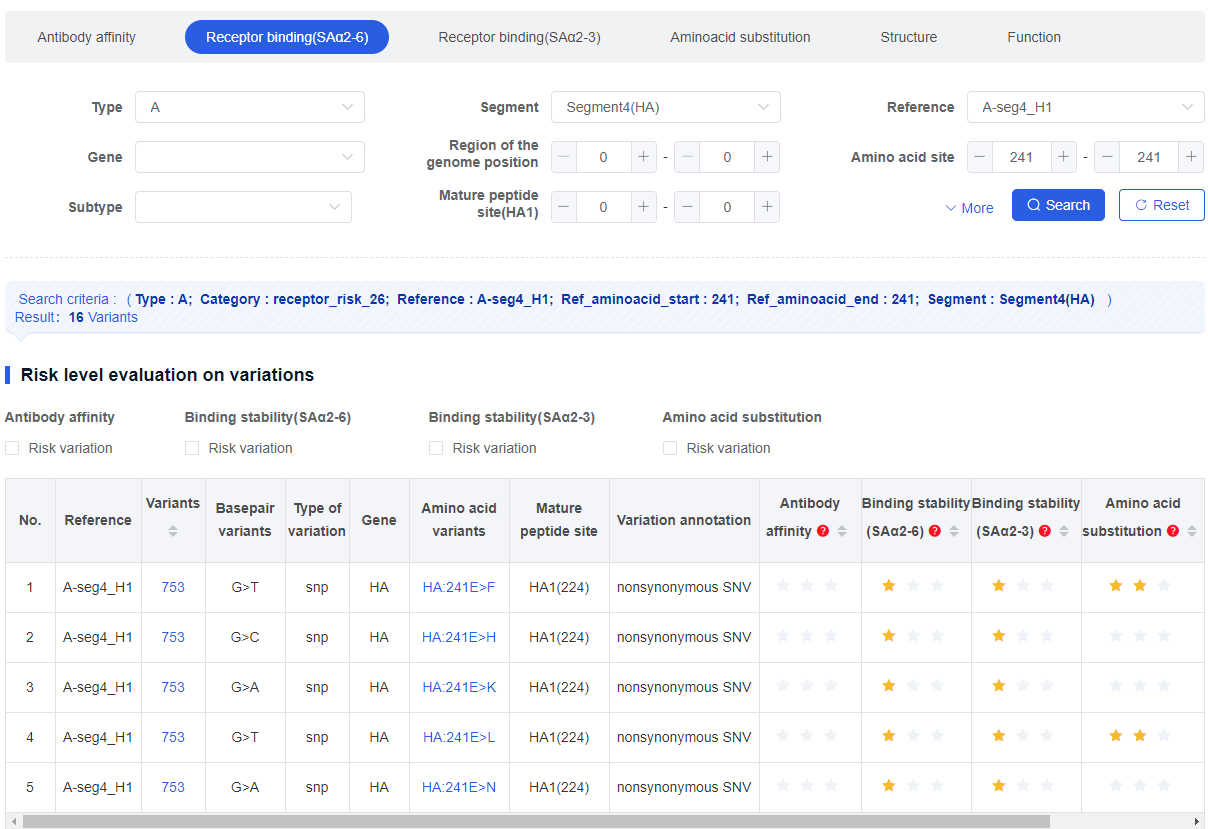


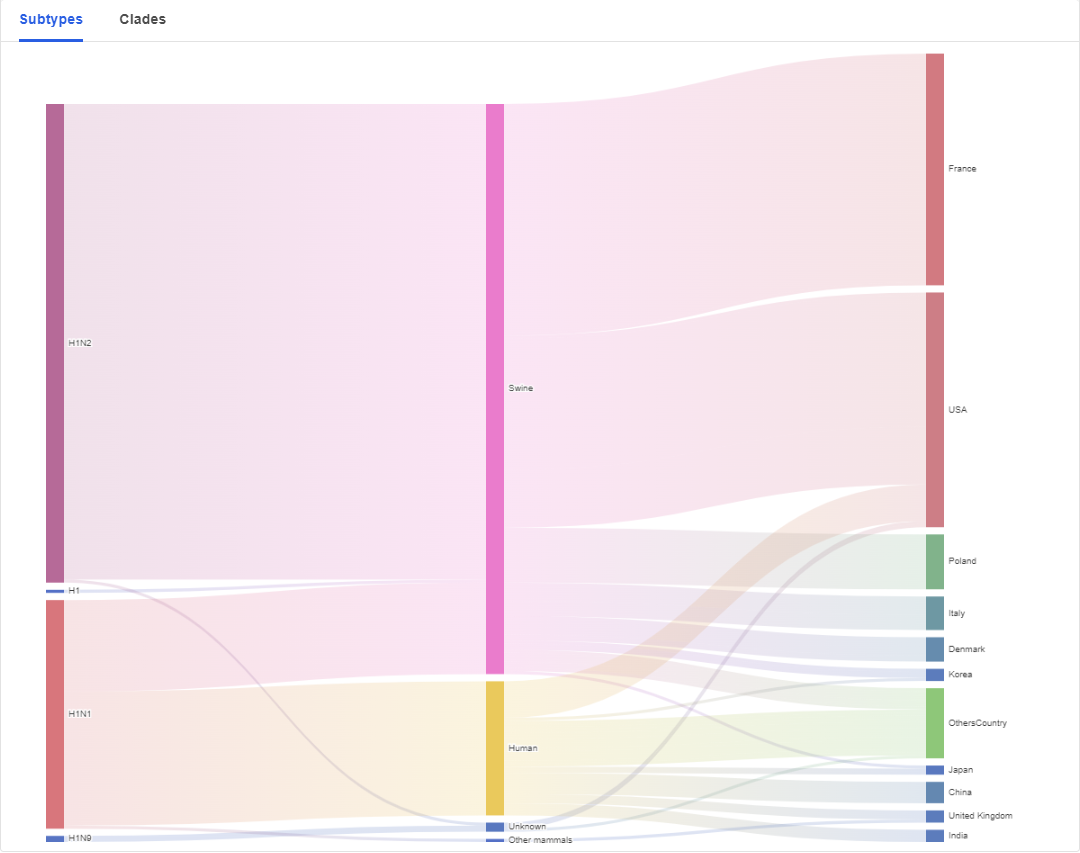


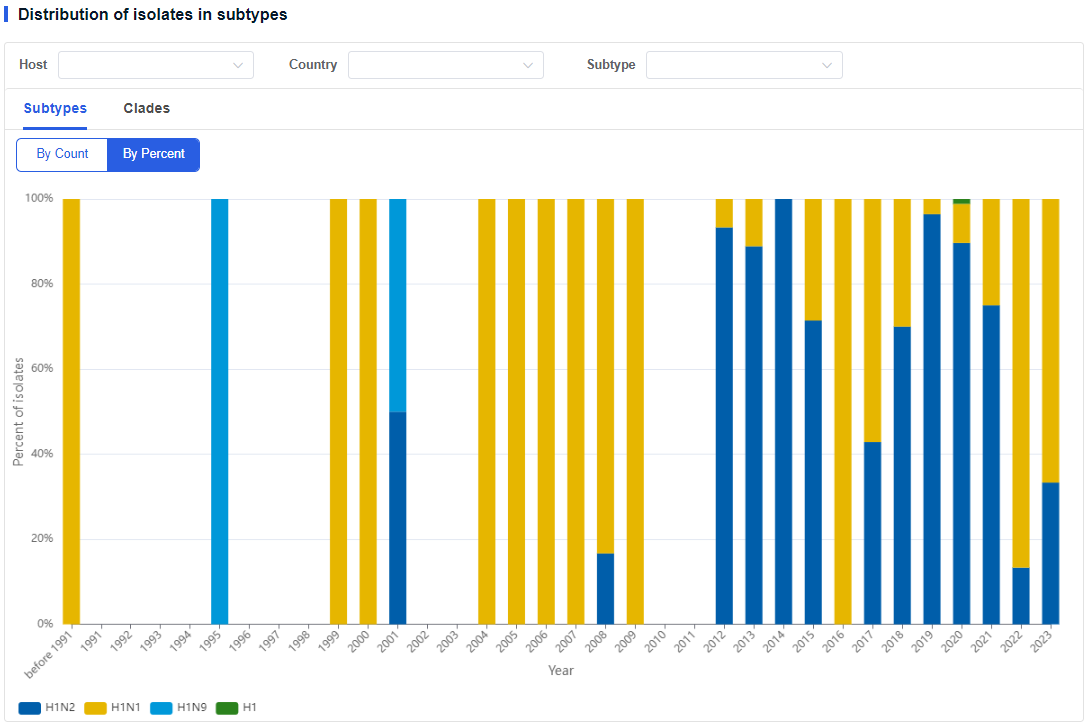


Next, the risk assessment and frequency heat map in the middle of “Segment” page (https://nmdc.cn/influvar/segment) can show the multi-dimensional assessment results of 19 amino acid variations. It is worth noting that there are 12 amino acid variations at locus 241 that indicate the affinity risk of sialic acid receptor α2,6 and sialic acid receptor α2,3. Four of these loci are "virtual mutations" that have not yet occurred.


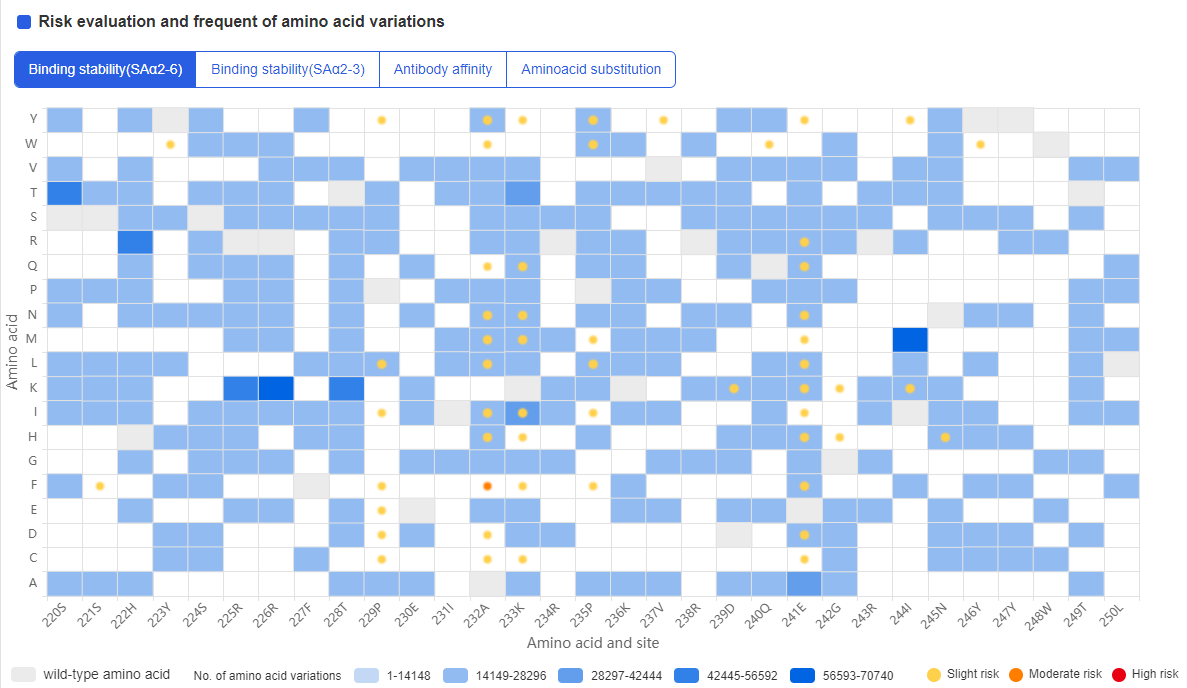


**Step4. Site Multidimensional Assessment Details:**

After summarizing the risk assessment for locus 241, we need to look in detail at the impact of each amino acid variant on multidimensional parameters. For the convenience of the user, we provide a variety of navigation options. You can click on the site (https://nmdc.cn/influvar/) from the left sidebar of the 3D map query module on the homepage, or direct navigation through specific amino acid variations in tables on the “Risk evaluation” page. In addition, enter the site location on the “variation” page and click on the specific amino acid variation (https://nmdc.cn/influvar/variation) in the table, you can go to the relevant detailed evaluation page.

The detailed evaluation page provides specific parameters for multi-dimensional evaluation, including lineage distribution, host distribution, temporal distribution, effects of variation on neutralizing antibody, affinity of sialic acid receptor α2,6 and sialic acid receptor α2,3, and antigen epitope. All these results are free for download. From the specific evaluation parameters, we can see that the E241k variation may have a greater impact on the antigenic epitope.


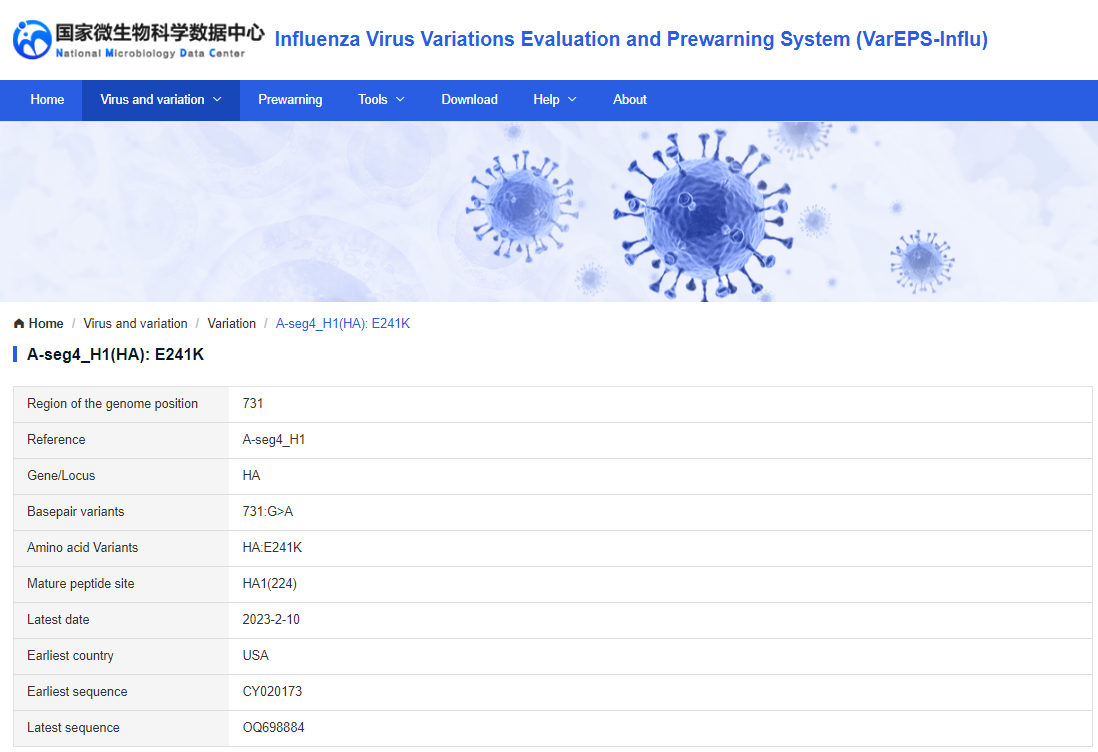


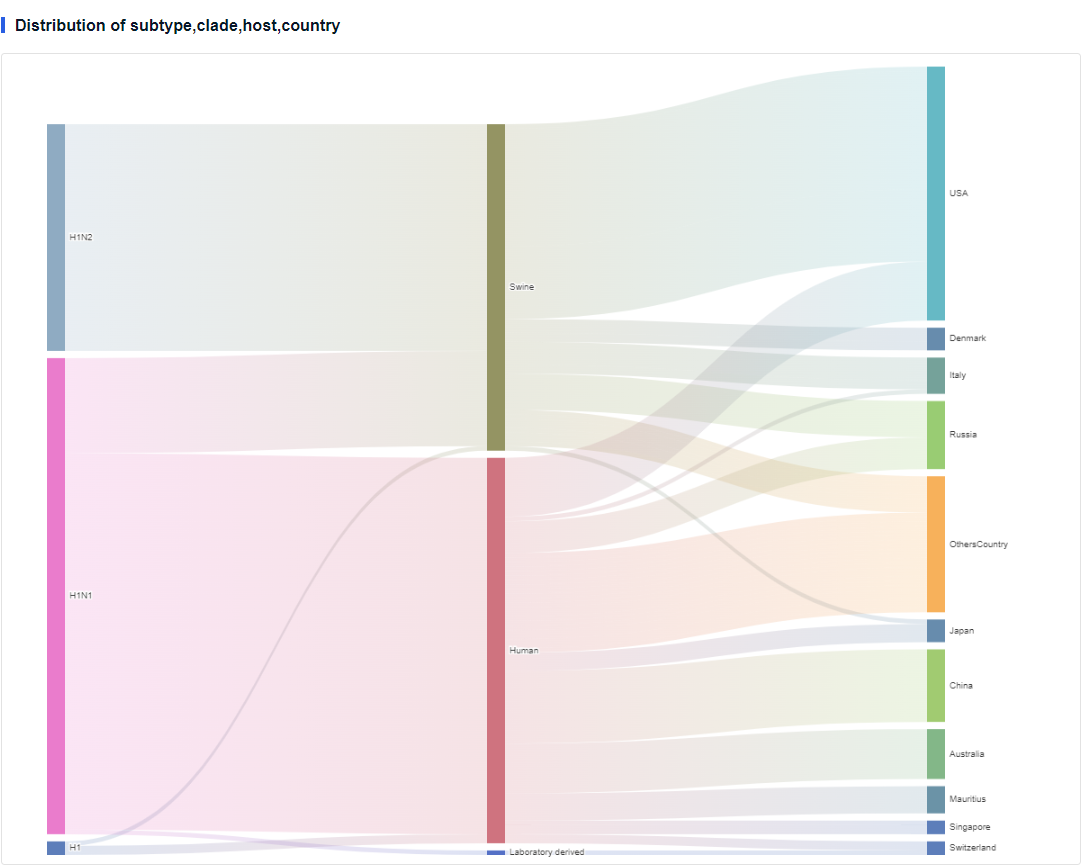


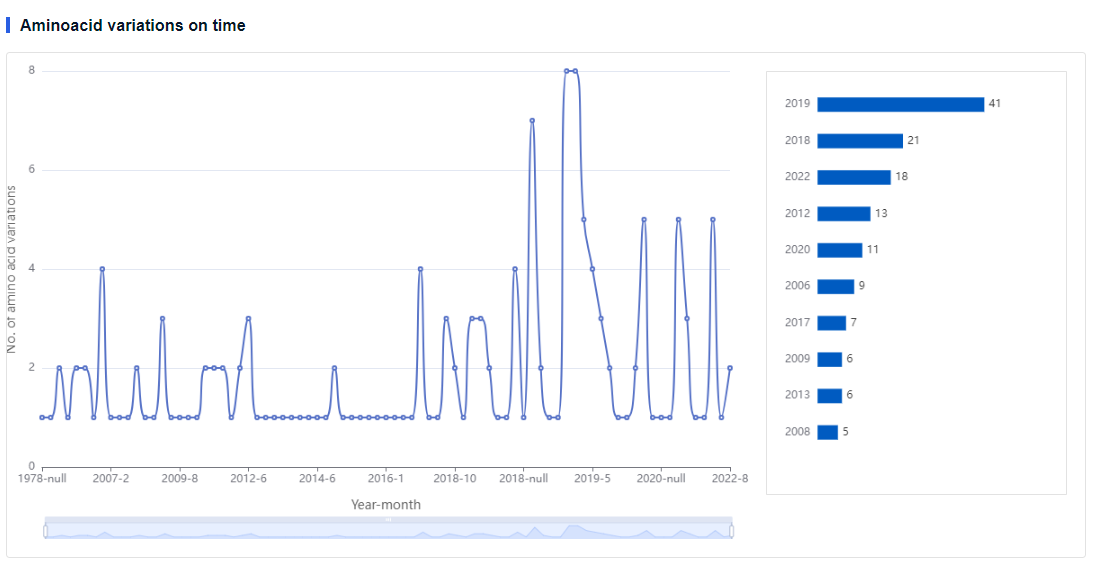


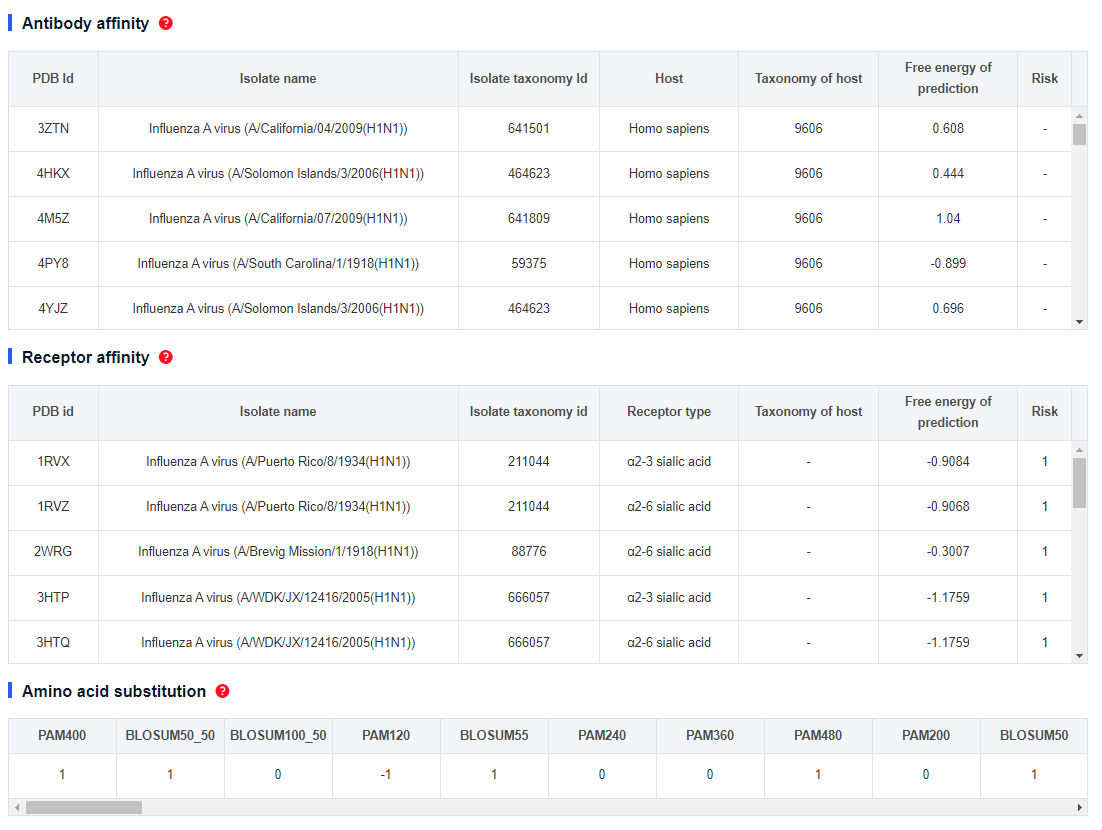


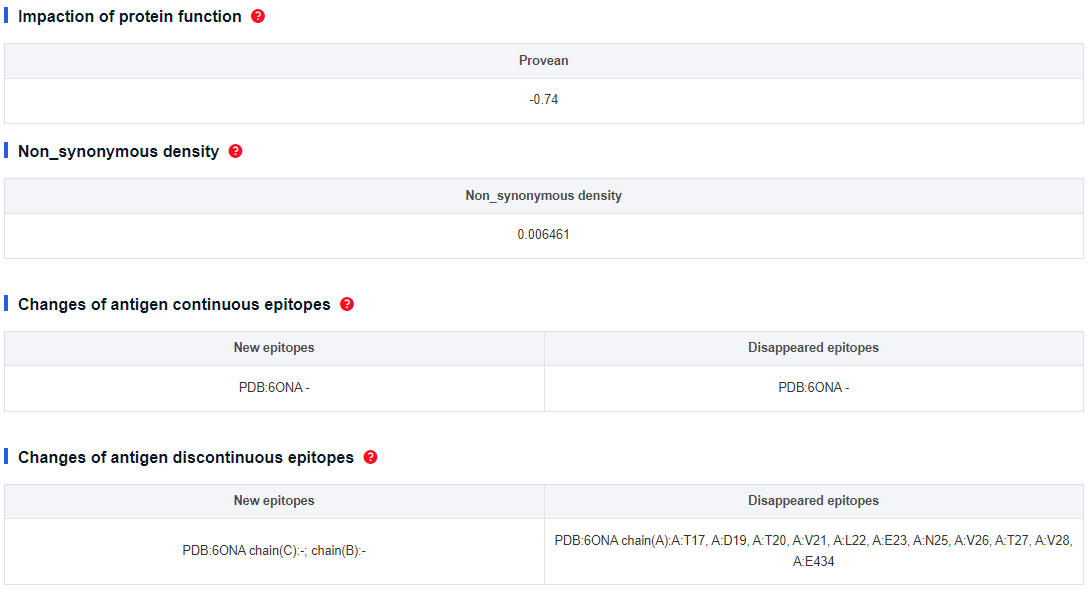


In summary, we used epidemiological data to identify three rapidly growing sites in the sequence of H1N1 human influenza viruses. Through multi-dimensional assessment, 241 sites were detected and range of interested sites was narrowed. Multidimensional analysis showed that the 12 variants at locus 241 may enhance the receptor-binding ability of influenza virus, thus improving the transmission ability of the virus. And some amino acid variants may have a greater impact on the epitope.These results can provide potential research clues and data supports for the experimental teams.
